# Early skeletal muscle loss during target therapy is a prognostic biomarker in metastatic renal cell carcinoma patients

**DOI:** 10.1038/s41598-017-07955-6

**Published:** 2017-08-08

**Authors:** Weijie Gu, Junlong Wu, Xiaohang Liu, Hailiang Zhang, Guohai Shi, Yao Zhu, Dingwei Ye

**Affiliations:** 1 Department of Urology, Fudan University Shanghai Cancer Center, Shanghai, People’s Republic of China; 2 Department of Oncology, Shanghai Medical College, Fudan University, Shanghai, People’s Republic of China; 3 Department of Radiology, Fudan University Shanghai Cancer Center, Shanghai, People’s Republic of China

## Abstract

Skeletal muscle depletion is common in patients with advanced cancer and may be associated with a poor outcome. To investigate whether the changes in skeletal muscle in metastatic renal cell carcinoma (mRCC) patients receiving targeted therapy are associated with clinical outcome, we undertook an observational cohort study using data from a number of randomized clinical trials previously conducted at the Fudan University Shanghai Cancer Center. The muscle mass was evaluated by comparing computed tomography images obtained at baseline with those obtained after 3–4 months of treatment. A total 101 patients were included in the study. The mean skeletal muscle area reduced from 41.6 cm^2^/m^2^ to 39.9 cm^2^/m^2^ after 3–4 months of targeted therapy. In multivariable analyses adjusted for the number of baseline covariates, muscle loss ≥5% was shown to be a significant prognostic factor for both progression-free (hazard ratio [HR]: 1.744, 95% confidence interval [CI]: 1.077–2.826, P = 0.024) and overall survival (HR: 2.367, 95%CI: 1.253–4.469, P = 0.008). The addition of muscle loss to the Heng model significantly improved its discriminative ability. In summary, early skeletal muscle loss is frequently observed in mRCC patients and can add prognostic information to current clinical risk scores.

## Introduction

Cachexia is a complex metabolic syndrome that typically occurs in cancer and other chronic diseases^1^. Cachexia is defined as at least a 5% loss of body weight or weight loss greater than 2% in individuals already showing depletion according to current body weight and height (body mass index [BMI] < 20 kg/m^2^) or skeletal muscle mass (sarcopenia)^2^. Cachexia is commonly identified in advanced and metastatic renal cell carcinoma (RCC) patients, and it can indicate a paraneoplastic syndrome^3^. Cachexia-like symptoms have been shown to be an independent biomarker of a worse prognosis in patients with early stage RCC^4^. The management of cancer-related cachexia remains a complex challenge; biomarkers that help to accurately diagnose cachexia in early stage patients, and predict progression, are urgently required.

Weight loss is commonly observed in cancer patients and is a classic diagnostic criterion for cachexia. It is generally accepted that the weight loss associated with advanced malignant diseases involves the loss of fat and lean body mass, especially skeletal muscle. Skeletal muscle wasting, termed sarcopenia, has been identified as an important independent prognostic factor in several tumor types^5–8^. Sarcopenia is also predictive of severe treatment toxicity in patients receiving chemotherapy and targeted therapy^9–11^. Computed tomography images that are available in patient’s medical record allow us to move away from overall body weight and BMI to being able to do multiple highly precise and specific measures of individual body components.

Although cross-sectional studies in metastatic RCC patients have shown that weight and tissue loss is specific to anti-angiogenic therapies^12^, few studies have investigated the prognostic value of longitudinal changes of body composition over time in metastatic RCC patients receiving targeted therapy.

Only very recently have we appreciated that the change of muscle mass over time is highly important^13^, but we did not know this for renal cell carcinoma. We decided to evaluate muscle mass alterations in metastatic RCC patients after 3–4 months of treatment, and to investigate any correlations with survival or treatment toxicity, using computed tomography (CT) images obtained during a number of previous phase II and III clinical trials.

## Subjects and Methods

### Study design

A retrospective observational study was performed using patient data from several prospective phase II and III clinical trials performed at the Fudan University Shanghai Cancer Center (NCT00706706, NCT00586105, NCT00720941, NCT01147822, NCT00920816, NCT02072031, NCT01829841, NCT01491672 and ChiCTR-ONRC-12002088). Most of the enrolled patients fulfilled the eligibility criteria: clear cell carcinoma; Eastern Cooperative Oncology Group (ECOG) performance status of 0–1; treated with targeted therapy for >3 months; treatment charts recording weight and height available; patients in a clinically stable condition without severe comorbidities; and no limitations on food access or intake. All of the patients included in the final study had at least 2 abdominal CT scans.

All of the patients gave written informed consent for the original phase II and III clinical trials. The original clinical trials and our subsequent analysis of the patients’ clinical data and CT images received approval from the Fudan University Shanghai Cancer Center institutional review board and all experiments were carried out in accordance with approved guidelines of Fudan University.

### Imaging and assessment of muscle mass

Weight and height were measured by trained study nurses using a calibrated stadiometer during trial visits. Patients’ BMI was calculated using the formula:

$${\rm{BMI}}={\rm{weight}}/{{\rm{height}}}^{2}.$$

The patients’ body composition and features were evaluated using the routine clinical trial CT images. To determine the effects of treatment on muscle mass, the CT images obtained at baseline were compared with those obtained after 3–4 months of targeted therapy. The baseline CT scans were all performed in the 2 weeks prior to targeted therapy initiation. The median time (±standard error) between the 2 CT scans was 107 ± 12 days. An experienced radiologist (X.L), who was blind to other variables and patient outcome, performed the image analysis.

The third lumbar vertebra (L3) was chosen as a landmark because it has been shown to be linearly correlated with whole-body skeletal muscle mass and adipose tissue mass^14^. The L3 region contains the psoas, paraspinal, and abdominal wall muscles. CT images were analyzed using ImageJ software (http://rsb.info.nih.gov/ij/), as previously described^15, 16^. Pre-established Hounsfield unit (Hu) tissue thresholds −29 to +150 and −190 to −30 were used for the skeletal muscle and adipose tissue analyses respectively^14, 17^. Muscle, visceral adipose and subcutaneous adipose areas (cm^2^) were computed automatically. Muscle density were measured using the muscle radiation attenuation rate (in Hu) because of its prognostic value in metastatic RCC patients^18^.

As in previous studies, muscle area was normalized for stature to account for BMI, and is expressed in units of cm^2^/m^2^ ^19^.

Using X-tile software^20^, patients were split into two groups based on their skeletal muscle index changes: group one included patients with the highest muscle loss ≥5%; and group two included patients with lower muscle loss <5% and those who gained muscle.

Sarcopenia at baseline was also considered in our study. Cut-off values based on the L3 skeletal muscle mass index proposed by Zhang *et al*.^21^ were used: <40.8 cm^2^/m^2^ for males and <34.9 cm^2^/m^2^ for females. The cutoff values for sarcopenia were obtained from a cohort of cancer patients in China from well powered large samples.

### Other assessment and outcomes

Reports of treatment toxicity between the two CT scans were collected from the patients’ records. Toxicity was classified and graded according to the Common Terminology Criteria for Adverse Events (CTCAE) v4.0.

Dose-limiting toxicity (DLT) was defined as any toxicity leading to a dose reduction, temporary or permanent discontinuation of treatment^11^. DLTs occurring during the first 3 months’ treatment were recorded for the present study.

Overall survival (OS) was defined as the time from targeted agent administration to the date of death or last contact. Progression-free survival (PFS) was defined as the time from treatment administration to the first documentation of disease progression, death from any cause, or last visit. Disease progression was determined on the basis of clinical examinations and radiological imaging using the Response Evaluation Criteria in Solid Tumors v1.1.

### Statistical analysis

Continuous variables are expressed as means ± standard deviation and categorical data are presented as proportions. The Student’s t-test and χ^2^ test were used for comparisons of continuous and categorical variables, respectively. Spearman’s rho test was used to evaluate the correlation between weight changes and muscle changes.

Survival curves were estimated using the Kaplan-Meier method, and differences between groups were evaluated using the log-rank test. Univariate and multivariate Cox proportional hazards models were used to test for associations between the investigated variables and PFS and OS. For body composition analyses, the linearity of risk was tested using restricted cubic splines, which enabled us to determine whether the non-linear component was statistically significant. To determine the incremental prognostic value of muscle loss on patient prognosis, Harrell’s concordance index (C index) was used to evaluate the discriminatory power of the Cox models. The likelihood ratio χ² test for nested models was used to assess whether additional variables added predictive value to the baseline models.

All statistical analyses were performed using R v3.0.1 (the R Project for Statistical Computing, Vienna, Austria, www.r-project.org). Two-tailed tests were used and statistical significance was defined as P < 0.05.

## Results

### Patient characteristics

**Figure 1:**
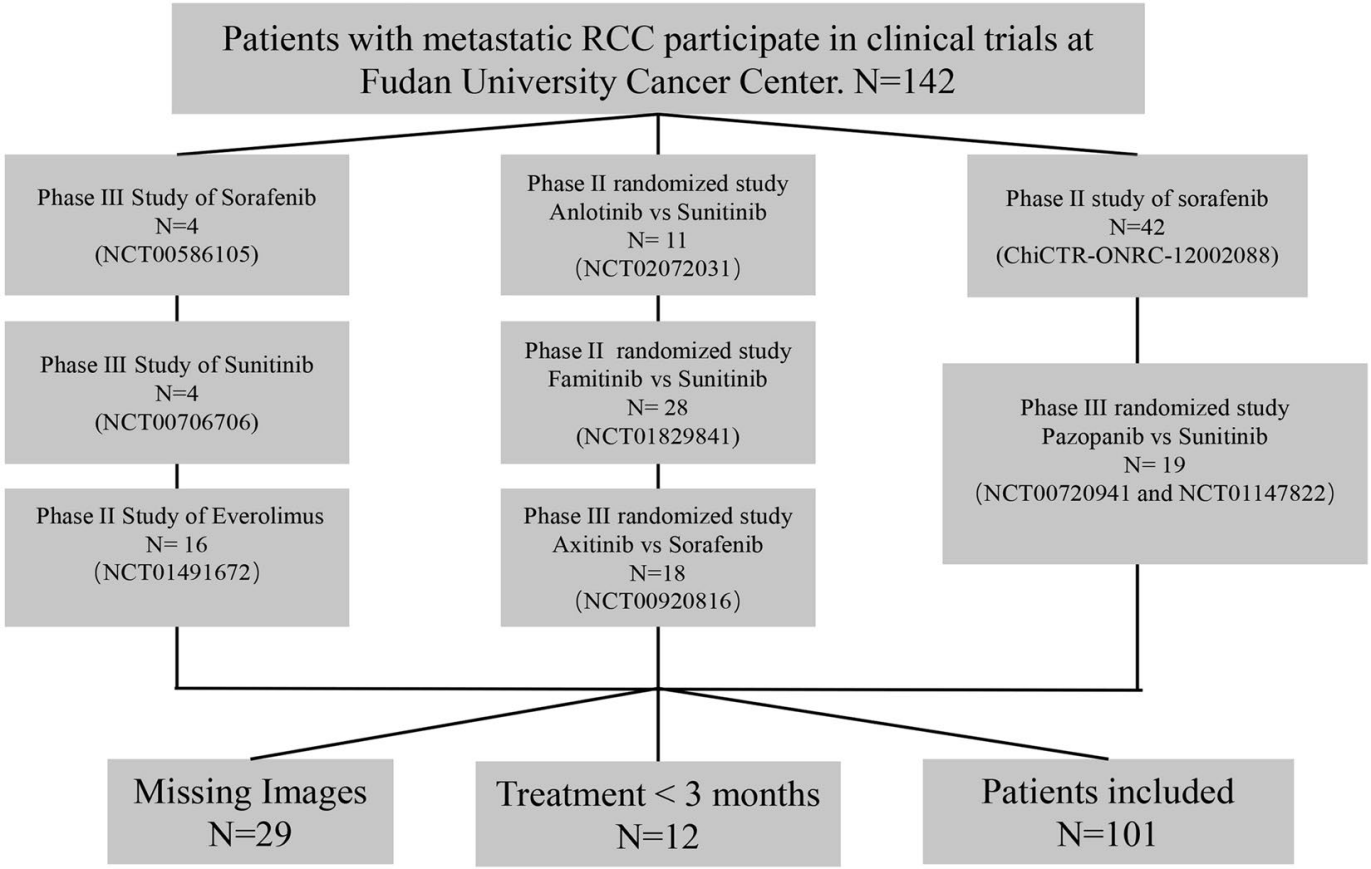
Patient inclusion schema.

From May 2008 to June 2014, 142 patients with metastatic RCC were enrolled in clinical trials at Fudan University Cancer Center. Of these, 101 patients were eligible for inclusion in the present study (Fig. 1). Exclusion criteria were loss to follow-up or no abdominal CT scan at either baseline or after 3–4 months of targeted therapy.

Clinical characteristics of the patient population are shown in Table 1; most patients (90.1%) had a good performance status (ECOG = 0). Using the Heng risk score^22^, 25.74% of the patients were classified as being low risk (no risk factors, 26 patients), 67.33% were classified as being intermediate risk (1–2 risk factors, 68 patients), and 6.93% were classified as being high risk (>2 risk factors, 7 patients). Patients were treated with tyrosine kinase inhibitors and mTOR inhibitors (sunitinib, n = 30; sorafenib, n = 45; pazopanib, n = 9; fabitinib, n = 10; axtinib, n = 4 and everolimus, n = 3). Seventy-four patients (73.2%) received targeted therapy as first-line treatment. Although the baseline body weight, visceral adipose tissue index, and subcutaneous adipose tissue index tended to be lower in patients which experienced muscle loss, no statistically significant associations were detected.

**Table 1:** Baseline Clinical Characteristics.

	All N = 101	Muscle loss <5% N = 46	Muscle loss ≥5% N = 55	*P*-value
Age		59.6 (12.8)	59.1 (13.9)	0.852
Gender				0.854
Male	65 (64.4)	29 (63.1)	36 (65.5)	
Female	36 (35.6)	17 (36.9)	19 (34.5)	
ECOG				0.424
0–1	91 (90.1)	43 (93.5)	48 (87.3)	
>1	10 (9.9)	3 (6.5)	7 (12.7)	
Heng risk score				0.609
Low	26 (25.7)	14 (30.4)	12 (21.8)	
Intermediate	68 (67.3)	29 (63.1)	39 (70.9)	
High	7 (7.0)	3 (6.5)	4 (7.3)	
Surgery				0.142
Yes	92 (91.1)	44 (95.6)	48 (87.3)	
No	9 (8.9)	2 (4.4)	7 (12.7)	
Drugs				0.593
Sunitinib	30 (29.7)	12 (26.1)	18 (32.7)	
Sorafenib	45 (44.6)	23 (50.0)	22 (37.3)	
Others	26 (25.7)	11 (23.9)	15 (30.0)	
Diabetes				0.082
Yes	7 (6.9)	3 (6.5)	4 (7.3)	
No	94 (93.1)	43 (93.5)	51 (92.7)	
NLR				0.073
<5	83 (82.2)	41 (89.1)	42 (76.4)	
≥5	18 (17.8)	5 (10.9)	13 (23.6)	
Albumin, m/l				0.497
<35	12 (11.9)	4 (8.7)	8 (14.6)	
≥35	89 (88.1)	42 (91.3)	47 (85.4)	
Creatinine clearancerate	79.5 (8.6)	78.2 (8.6)	80.6 (8.4)	0.154
Height, cm	166.3 (7.4)	166.7 (6.8)	166.2 (8.1)	0.764
Weight, Kg	63.8 (12.1)	66.2 (11.3)	62.6 (12.9)	0.137
BMI, Kg/m^2^	22.9 (3.4)	23.5 (3.3)	22.4 (3.6)	0.146
Skeletal muscle index, cm^2^/m^2^	41.8 (7.6)	41.5 (7.2)	42.0 (8.0)	0.720
Skeletal muscle density, Hu	26.9 (2.2)	27.0 (2.6)	26.9 (1.9)	0.944
Visceral adipose tissue index, cm^2^/m^2^	34.6 (22.3)	36.3 (20.2)	32.6 (24.9)	0.421
Subcutaneous tissue index, cm^2^/m^2^	38.5 (22.7)	40.1 (22.4)	37.2 (25.9)	0.544
Sarcopenia	36 (35.6)	17 (36.9)	19 (34.5)	0.801
Non sarcopenia	65 (64.4)	29 (63.1)	36 (65.5)	

Abbreviation: BMI body mass index; NLR: Neutrophil to lymphocyte ratio; ECOG Eastern Cooperative Oncology Group Performance Status.

### Changes in skeletal muscle and body weight

The mean skeletal muscle index in the cohort reduced from 41.6 cm^2^/m^2^ to 39.9 cm^2^/m^2^ after 3–4 months of targeted therapy. A reduced skeletal muscle index was observed in 63 patients (62.4%), with a mean decrease of 4.3 cm^2^/m^2^. The mean skeletal muscle density in the cohort decreased from 35.8 Hu to 33.9 Hu.

**Figure 2:**
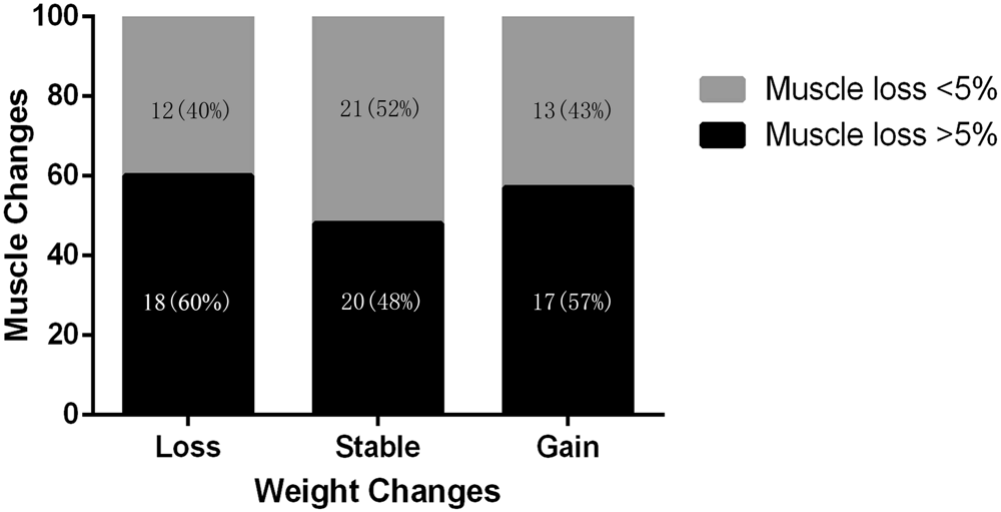
Correlations between muscle area changes and body weight changes. Loss means weight loss ≥5%, stable means weight loss <5% and weight gain <2%, gain means weight gain >2%.

Weight loss ≥5% was experienced by 30 patients during targeted therapy, and 12 patients experienced muscle loss <5%. Among the 71 patients with a stable or gain weight (weight loss <5%), about half experienced significant muscle loss ≥5. Similarly, 36 (35.6%) patients were sarcopenic at baseline, and 17 (47.2%) experienced muscle loss <5%. Among the non-sarcopenic patients, 29 patients (44.6%) experienced muscle loss <5%. No correlations between muscle area changes and body weight changes were observed (Spearman’s rho, 0.32; P = 0.07; Fig. 2).

### Muscle loss and outcomes

**Figure 3:**
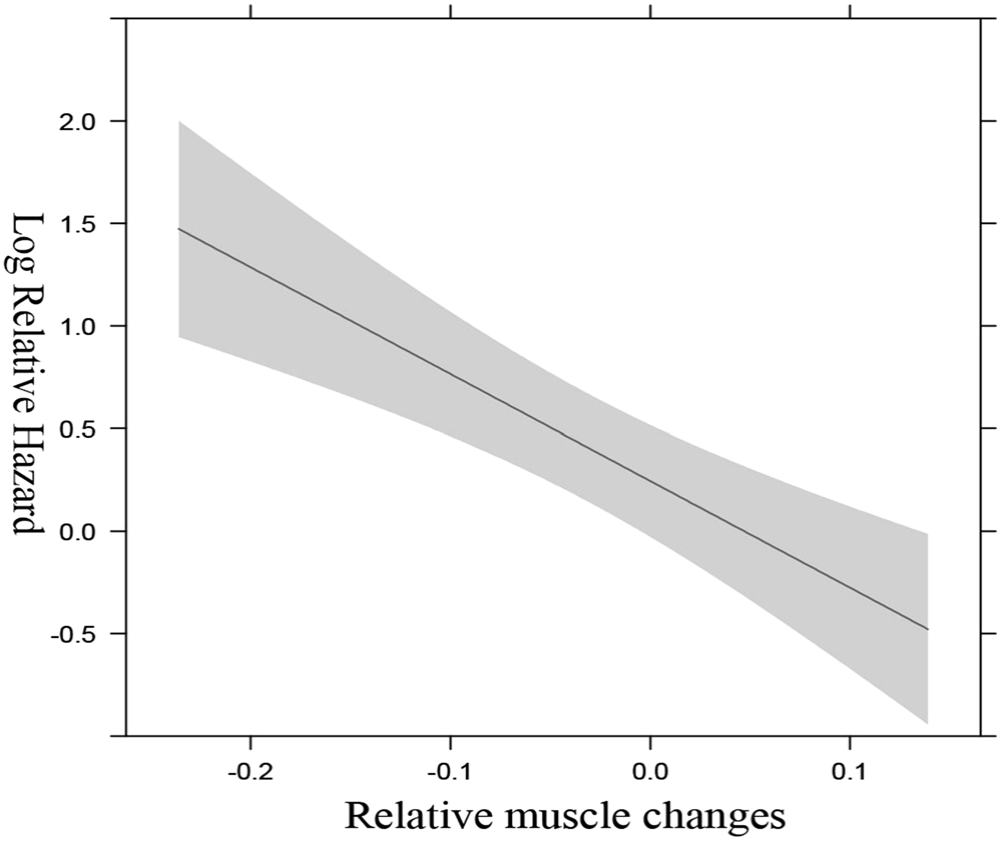
Relationship between relative changes in skeletal muscle loss and total mortality using restricted cubic splines.

The median follow-up period was 30.8 months (95% confidence interval (CI): 24.1–37.4 months); during that time 56 patients (55.4%) died. The median PFS and OS of all 101 metastatic RCC patients were 11.3 months and 25.9 months, respectively. Univariate Cox regression analyses indicated that skeletal muscle was associated with a shorter PFS (hazard ratio (HR): 1.745, 95% CI: 1.102–2.762, P = 0.018) and OS (HR: 2.186, 95% CI: 1.209–3.952, P = 0.010; Table 2). However, the changes of body weight, visceral adipose tissue index, and subcutaneous adipose tissue index did not show statistically significant associations with survival. In multivariable analyses adjusted for the number of baseline covariates, multivariate Cox survival analysis indicated that skeletal muscle loss ≥5% remained a significant prognostic factor for poor PFS (HR: 1.744, 95% CI: 1.077–2.826, P = 0.024) and OS (HR: 2.367, 95% CI: 1.253–4.469, P = 0.008; Table 3). After multivariable adjustment, each unit increase in muscle mass alteration was associated with a HR for total mortality of 0.964 (95% CI 0.948–0.980, P < 0.001). The relationship between changes in skeletal mass loss and total mortality with muscle loss was linear (Fig. 3).

**Table 2:** Univariate Survival analysis.

	Progression free survival		Overall survival	
	HR	*P*-value	HR	*P*-value
Age	0.989 (0.972–1.006)	0.214	0.985 (0.964–1.005)	0.145
Gender				
Male	1.0		1.0	
Female	0.890 (0.559–1.657)	0.890	0.787 (0.442–1.399)	0.414
Surgery				
Yes	1.0		1.0	
No	0.556 (0.250–1.234)	0.149	0.554 (0.249–1.236)	0.149
Heng				
Low	1.0		1.0	
Intermediate	3.082 (1.443–6.579)	0.004	4.898 (1.929–12.436)	0.001
High	4.920 (1.580–15.314)	0.006	11.360 (3.201–40.306)	<0.001
NLR				
<5	1.0		1.0	
≥5	1.326 (0.700–2.512)	0.387	1.471 (0.734–2.949)	0.276
BMI, Kg/m^2^				
<23	1.0		1.0	
≥23	0.879 (0.564–1.369)	0.568	0.618 (0.353–1.082)	0.092
Albumin, m/l				
<35	1.0		1.0	
≥35	0.570 (0.289–1.123)	0.104	0.340 (0.164–0.708)	0.004
CCI	1.063 (0.726–1.556)	0.755	1.199 (0.766–1.876)	0.427
Baseline skeletal muscle index	1.005 (0.977–1.034)	0.750	0.980 (0.944–1.017)	0.112
Baseline skeletal muscle density	0.997 (0.954–1.043)	0.910	0.988 (0.948–1.030)	0.579
Visceral adipose change	0.996 (0.989–1.002)	0.210	1.002 (0.994–1.010)	0.662
Subcutaneous adipose change	0.997 (0.990–1.003)	0.317	0.995 (0.987–1.003)	0.193
Weight change	0.969 (0.878–1.069)	0.526	1.035 (0.917–1.169)	0.577
Skeletal Muscle change				
Loss <5%	1.0		1.0	
Loss ≥5%	1.745 (1.102–2.762)	0.018	2.186 (1.209–3.952)	0.010
Baseline sarcopenia				
No	1.0		1.0	
Yes	1.426 (0.880–2.310)	0.150	1.155 (0.645–2.068)	0.628

Abbreviation: BMI: body mass index NLR: Neutrophil to lymphocyte ratio CCI: Charlson comorbidity index.

**Table 3:** Multivariate Survival Analysis.

	Progression free survival		Overall survival	
	HR	*P*-value	HR	*P*-value
Age	0.988 (0.969–1.008)	0.163	0.984 (0.963–1.005)	0.984
Gender				
Male	1.0		1.0	
Female	0.788 (0.464–1.004)	0.081	0.461 (0.232–0.916)	0.027
Heng				
Low	1.0		1.0	
Intermediate	1.428 (1.019–3.281)	0.044	4.684 (1.819–12.061)	0.002
High	3.125 (1.278–11.122)	0.025	12.574 (3.331–47.466)	0.001
NLR				
<5	1.0		1.0	
≥5	1.280 (0.664–2.467)	0.461	1.271 (0.579–2.703)	0.534
BMI, Kg/m^2^				
<23	1.0		1.0	
≥23	0.757 (0.460–1.345)	0.499	0.618 (0.326–2.703)	0.139
CCI	0.930 (0.613–1.411)	0.732	0.965 (0.580–1.608)	0.893
Skeletal muscle change				
Loss <5%	1.0		1.0	
Loss ≥5%	1.744 (1.077–2.826)	0.024	2.367 (1.253–4.469)	0.008

Abbreviation: BMI: body mass index NLR: Neutrophil to lymphocyte ratio CCI: Charlson comorbidity index.

**Figure 4:**
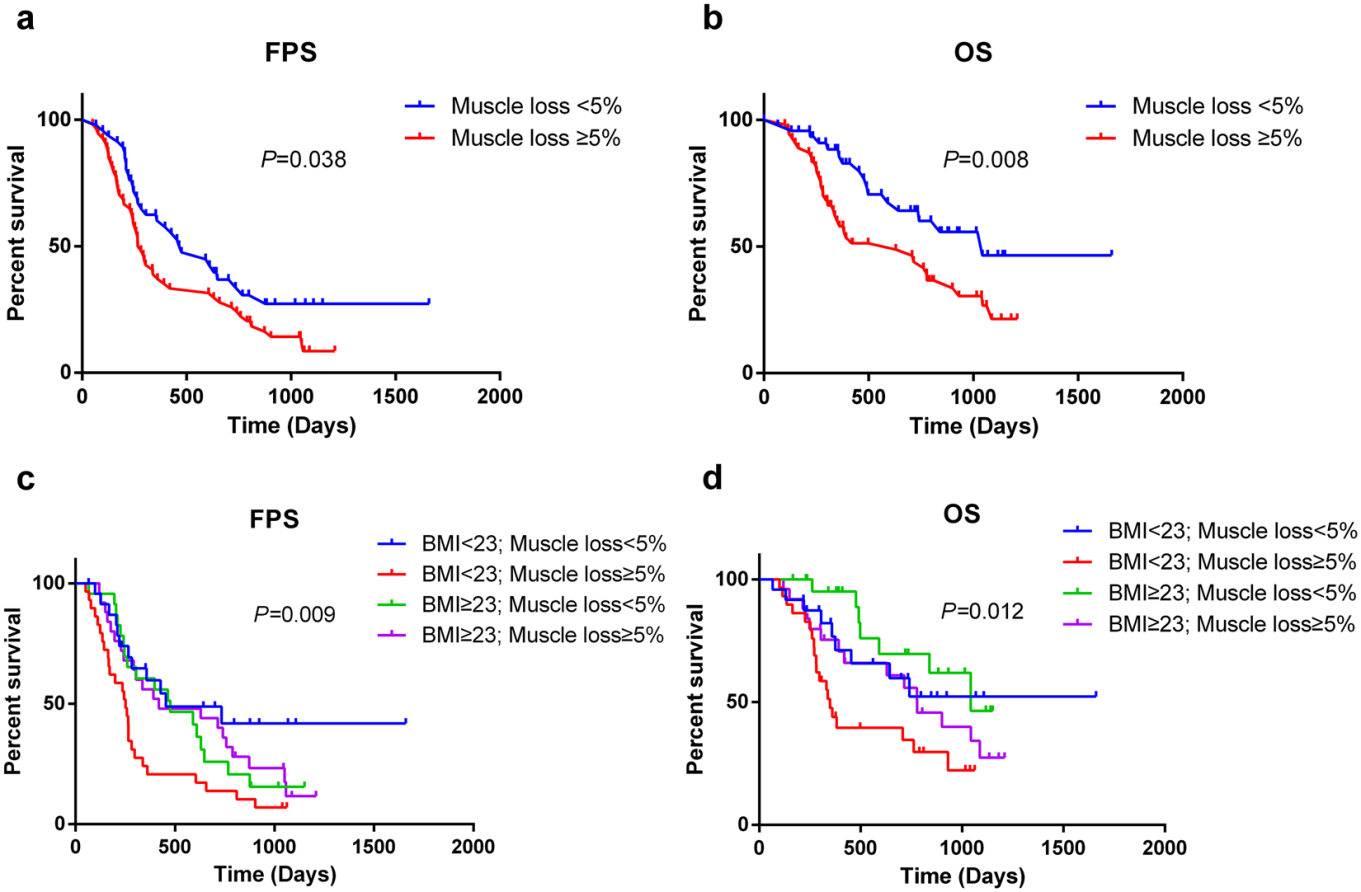
Kaplan-Meier curves for comparison of survival between patients with muscle loss (muscle loss ≥5%)and those with stable muscle (muscle loss <5%). (**a**) Progression free survival (log-rank P = 0.038). (**b**) Overall survival (log-rank P = 0.008). Adding BMI to separate the patients into 4 goups. (**c**) Progression free survival (log-rank P = 0.009). (**d**) Overall survival (log-rank P = 0.012).

Kaplan-Meier curves indicated that the median OS of patients with a stable skeletal muscle (muscle loss <5%) was 1.6-fold greater than the OS of patients with muscle loss (34.7 months vs 20.9 months, P = 0.008, log-rank); similar results were found for the median PFS (15.8 months vs 9.1 months, P = 0.035, log-rank; Fig. 4a,b). Taking into different basal nutrition status, the predictive value of muscle loss was also seen in patients with different BMI groups (Fig. 1c,d). Adding BMI will separate the muscle loss into 2 groups, with the median OS periods ranging from 11.6 months for muscle loss/low BMI to 25.9 months for muscle loss/high BMI and the median PFS periods ranging from 8.4 months for muscle loss/low BMI to 13.9 months for muscle loss/high BMI.

The association between sarcopenia at baseline and OS was also not detected (sarcopenic vs non-sarcopenic; HR: 1.155, 95% CI: 0.645-2.068, P = 0.628). Moreover, baseline skeletal muscle index and radiodensity were not also significant prognostic factors for OS.

The addition of muscle loss to the Heng model significantly improved its discriminative ability (C index increased from 0.691 to 0.729). When substituted for performance status, a factor of the from Heng risk model, muscle loss increased the C index (from 0.691 to 0.712) and discriminative ability of the model. Furthermore, the muscle loss also had higher predictive power than body weight changes, when adding them to Heng model respectively (C index was 0.729 vs 0.697).

### Muscle loss and toxicity

Thrombocytopenia (n = 11) and hand-foot syndrome (n = 7) were the most common grade 3 and 4 toxicities (Table 4). No significant differences in grade 3 and 4 toxicity were observed between patients who maintained/gained skeletal muscle and those who suffered muscle loss. Similarly, no differences in gastrointestinal toxicity were observed between the groups.

**Table 4:** Common adverse events collected during targeted therapy*.

	Muscle loss <5%	Muscle loss ≥5%	*P*-value
Hematological grade 1–2 toxicity			
Anemia	8	9	0.891
Neutropenia	9	15	0.356
Thrombocytopenia	7	8	0.925
Non-hematological grade 1–2 toxicity			
Fatigue	7	13	0.290
Nausea	5	11	0.211
Vomiting	2	4	0.536
Diarrhea	4	16	0.010
Hypertension	12	6	0.047
Hand-foot syndrome	13	7	0.051
Mucosal inflammation	6	4	0.334
Albuminuria	2	2	0.855
Increased creatinine	1	3	0.400
Weight loss	1	8	0.030
Liver dysfunction	4	6	0.711
Hematological grade 3-4 toxicity			
Anemia	1	1	0.898
Neutropenia	3	2	0.506
Thrombocytopenia	5	6	0.995
Non-hematological grade 3–4 toxicity			
Fatigue	2	3	0.798
Diarrhea	0	2	0.191
Hypertension	3	1	0.227
Hand-foot syndrome	4	3	0.523
Albuminuria	1	2	0.666
Liver dysfunction	2	2	0.855

*CTCAE 4.0.

Thirty-two (31.7%) patients experienced DLTs after 3 months targeted therapy, among whom 22 (40.0%) experienced muscle loss ≥5% and 10 (21.7%) experienced muscle loss <5%. No significant difference of muscle changes was observed between the patients with DLTs and those without DLTs (Table 5).

**Table 5:** Comparison of anthropometric parameters and body composition in function of dose-limiting toxicity.

Variable (SD)	Patients with dose-limiting toxicity	Patients who received the entire planned dose	*P*-value
Male	N = 23	N = 42	
Baseline BMI (kg/m^2^)	22.5 (3.2)	23.9 (2.8)	0.067
Weight change (kg)			0.384
Loss <5%	14	30	
Loss ≥5%	9	12	
Baseline skeletal muscle index (cm^2^/m^2^)	44.3 (6.4)	44.7 (7.7)	0.804
Skeletal muscle change			0.085
Loss <5%	7	22	
Loss ≥5%	16	20	
Female	N = 9	N = 27	
BMI (kg/m^2^)	20.7 (4.7)	22.9 (4.0)	0.258
Weight change (kg)			0.849
Loss <5%	6	21	
Loss ≥5%	3	9	
Baseline skeletal muscle index (cm^2^/m^2^)	32.4 (4.0)	37.1 (5.8)	0.093
Skeletal muscle change			0.335
Loss <5%	3	14	
Loss ≥5%	6	13	
Total	N = 32	N = 69	
BMI (kg/m^2^)	23.4 (3.4)	22.2 (3.4)	0.093
Weight change (kg)			0.243
Loss <5%	20	51	
Loss ≥5%	12	18	
Baseline skeletal muscle index (cm^2^/m^2^)	42.5 (7.8)	41.1 (7.5)	0.409
Skeletal muscle change			0.047
Loss <5%	10	36	
Loss ≥5%	22	33	

Abbreviation: BMI: body mass index.

## Discussion

This exploratory study provides important insights into the association between changes in muscle area and muscle density with metastatic RCC patient outcome. We observed a mean reduction in muscle area and muscle density of 1.71 cm^2^/m^2^ and 5.6 Hu after 3–4 months of targeted therapy. There were large variations in muscle area changes between patients, and muscle loss was frequently observed after 3–4 months of anti-cancer treatment (62.4% of the cohort experienced muscle loss). This corroborates the findings of previous studies that investigated muscle loss in metastatic colorectal and lung cancer patients receiving chemotherapy^13, 23^. To the best of our knowledge, ours is the first study to show that muscle loss is a significant prognostic factor for patients with metastatic RCC. We have also been able to show that muscle loss significantly improves the predictive ability of the Heng risk model (as shown by the increased the C index).

Changes in skeletal muscle index in metastatic RCC patients treated with targeted therapy were first reported by Antoun^12^, who observed a loss of muscle after the initiation of sorafenib treatment, and a loss of adipose tissue in long-term sorafenib-treated patients; a progressive loss of skeletal muscle was observed, with a mean decrease of 4.9% and 8.0% after 6 and 12 months treatment. This observation is of great importance; it shows that metastatic RCC patients have a high incidence of advanced muscle wasting associated with the use of targeted therapies that influence muscular protein anabolism. Our study using CT images extends these findings, and shows that as many as 62.4% of patients experience muscle loss (average decrease of 3.5%) after 3–4 months of targeted therapy treatment. Moreover, patients who experienced early stage muscle loss greater than 5% were at a significantly greater risk of mortality. The patients’ baseline Heng risk score, which is known to predict poor outcomes in metastatic RCC patients receiving first-line and second-line targeted treatment^22, 24^, did not affect the observed association between muscle loss and a poor outcome. Indeed, adding skeletal muscle area changes to the Heng model improved the predictive ability of the model.

In our study, treatment side effects were more frequently reported among patients with muscle loss, although no significant differences in grade 3 or 4 toxicity between muscle stable (muscle loss ≥5%) and muscle loss (muscle loss <5%) groups were found. However, patients with a stable skeletal muscle (muscle loss <5%) did show a slight improvement in self-reported mental attitude compared with other patients. Moreover, those patients are more likely to receive the entire planned dose than those with significant muscle loss. Previous studies indicated baseline skeletal muscle depletion predicted the DLTs in mRCC patients treated with targeted therapy^11, 25^. Our results showed significant more DLTs were observed in patients with the early stage skeletal muscle loss during the targeted therapy, which may translate into a significantly poorer PFS or poorer OS because of the decreased drug exposure.

Weight loss has been shown to be associated with a poor clinical outcome^26^. In our study, however, we did not observe a correlation between weight loss and muscle loss as well as the prognostic value. A possible explanation for this is that anti-cancer therapies may affect weight in several ways: anti-cancer drugs can inhibit angiogenesis, leading to the tumor shrinkage and a reversal of muscle loss; gastrointestinal toxicity can affect energy intake and cause weight loss, and exacerbate the progression of cancer-related cachexia; and side effects such as hypothyroidism can increase body weight due to edema, but this weight gain does not prevent progression to cachexia. Thus, the onset of muscle loss may be a more sensitive surrogate of oncologic cachexia than weight loss. Furthermore, the assessment of skeletal muscle area by diagnostic CT images is accurate, requires little time, and is almost cost free.

In our cohort of metastatic RCC patients, sarcopenia at baseline and muscle density changes were not associated with patient survival. This is in contrast to some previous studies^5, 18^. A possible explanation for this discrepancy is the cut-off levels that were used to define sarcopenia, and the small sample size of the study. Our study, however, indicated the change of skeletal muscle over time is a more sensitive biomarker than baseline sarcopenia.

Although the heterogeneous treatments and a small sample size in our study limit the power of the survival analyses, our study has several strengths. Unlike other studies of this type, patients and clinical data in our study were collected within prospective randomized clinical trials. Furthermore, the body composition data was blinded measured by an experienced radiologist. Our results indicate that early muscle loss in cancer patients can be detected, and that this associates with survival. Further prospective studies with larger metastatic RCC populations are required to confirm our findings, and to determine whether interventions aimed at preserving skeletal muscle during treatment can effectively improve patient outcome.

In conclusion, early muscle loss was frequently observed in metastatic RCC patients treated with targeted therapies, and this muscle loss was independently associated with a poor clinical outcome. The addition of muscle loss to the current Heng risk model improves the model’s prognostic value.
